# Incidence and characteristics of vitamin D deficiency rickets in New Zealand children: a prospective New Zealand paediatric surveillance unit study

**DOI:** 10.1186/1687-9856-2015-S1-P57

**Published:** 2015-04-28

**Authors:** Benjamin Wheeler, Nigel Dickson, Lisa Houghton, Leanne Ward, Barry Taylor

**Affiliations:** 1 Department of Women’s and Children’s Health, Dunedin School of Medicine, University of Otago, Dunedin, New Zealand; 2 Paediatric Endocrinology, Southern District Health Board, Dunedin, New Zealand; 3 New Zealand Paediatric Surveillance Unit, Department of Paediatrics and Child Health, Dunedin School of Medicine, University of Otago, Dunedin, New Zealand; 4 Department of Human Nutrition, University of Otago, Dunedin, New Zealand; 5 Department of Pediatrics, University of Ottawa, Ottawa, Ontario, Canada

## 

Vitamin D deficiency rickets is the most significant manifestation of vitamin D deficiency in growing children. Concerns have been raised in New Zealand (NZ), and worldwide, that cases continue to present, and may be possibly increasing. We undertook a prospective study to investigate the incidence and characteristics of vitamin D deficiency rickets in NZ children.

Prospective surveillance of Vitamin D Deficiency Rickets was conducted by the NZ Paediatric Surveillance Unit (NZPSU), for 36 months, from July 2010 – June 2013 inclusive. Inclusion criteria were: children aged <15 years with vitamin D deficiency rickets (defined by low 25-hydroxyvitamin D and elevated alkaline phosphatase levels, and/or radiological rickets).

58 children with confirmed vitamin D deficiency rickets were identified. Median age was 1.4 years (range 0.3 – 11), male gender 47%, 95% of children were born in NZ, as opposed to 22% of mothers. Overall annual incidence in those aged <15 years was 2.2/100,000, while incidence in the south of NZ peaked at 6.8/100,000. Overall NZ incidence in children aged <5 years was higher at 6.6/100,000. Skeletal abnormalities, poor growth and developmental delay were the most common presenting features, with hypocalcaemic convulsion in 16%. Key risk factors identified were: dark skin pigment, Indian/South Asian and African ethnicity, age ≤2 years, exclusive breast feeding, and southern latitude, particularly when combined with season (winter/spring).

Vitamin D deficiency rickets remains a health problem for New Zealand children, with significant associated morbidity. Public health policy, utilising infant supplementation, for at minimum the above identified risk factors, should be considered to reduce the incidence of this disease among those at high risk.

